# Molecular signatures associated with prostate cancer cell line (PC-3) exposure to inactivated Zika virus

**DOI:** 10.1038/s41598-019-51954-8

**Published:** 2019-10-25

**Authors:** Jeany Delafiori, Estela de Oliveira Lima, Mohamed Ziad Dabaja, Flávia Luísa Dias-Audibert, Diogo Noin de Oliveira, Carlos Fernando Odir Rodrigues Melo, Karen Noda Morishita, Geovana Manzan Sales, Ana Lucia Tasca Gois Ruiz, Gisele Goulart da Silva, Marcelo Lancellotti, Rodrigo Ramos Catharino

**Affiliations:** 1 Innovare Biomarkers Laboratory, School of Pharmaceutical Sciences, University of Campinas (UNICAMP), Campinas, São Paulo Brazil; 2 Medical School, São Paulo State University (UNESP), Botucatu, São Paulo Brazil; 3 School of Pharmaceutical Sciences, University of Campinas (UNICAMP), Campinas, São Paulo Brazil; 4 Department of Physiological Sciences, Piracicaba Dental School, University of Campinas (UNICAMP), Piracicaba, São Paulo Brazil; 5 Laboratory of Biotechnology, School of Pharmaceutical Sciences, University of Campinas (UNICAMP), Campinas, São Paulo Brazil

**Keywords:** Virology, Biotechnology

## Abstract

The recent outbreak of Zika virus (ZIKV) infection associated with microcephaly cases has elicited much research on the mechanisms involved in ZIKV-host cell interactions. It has been described that Zika virus impairs cell growth, raising a hypothesis about its oncolytic potential against cancer cells. ZIKV tumor cell growth inhibition was later confirmed for glioblastoma. It was also demonstrated that an inactivated ZIKV prototype (ZVp) based on bacterial outer membrane vesicles has antiproliferative activity upon other cancer cell lines, such as PC-3 prostate cancer cell. This study aims at understanding the pathways that might be involved with the antiproliferative effect of Zika virus against prostate cancer cells. A metabolomic approach based on high-resolution mass spectrometry analysis led to the identification of 21 statistically relevant markers of PC-3 cells treated with ZVp. The markers were associated with metabolic alterations that trigger lipid remodeling, endoplasmic reticulum stress, inflammatory mediators, as well as disrupted porphyrin and folate metabolism. These findings highlight molecular signatures of ZVp-induced response that may be involved on cellular pathways triggered by its antiproliferative effect. To our knowledge, this is the first reported metabolomic assessment of ZIKV effect on prostate cancer cells, a promising topic for further research.

## Introduction

The recent outbreak of Zika virus infection associated with microcephaly cases on South and Central America emerged the need for understanding the mechanisms involved in ZIKV-host cell interaction^1^. It was observed that the tropism of ZIKV to neuronal cells leads to consequent impairment of stem cells differentiation, neuronal growth, cellular cycle arrest, and cell death^2,3^. Zika virus-mediated cell death has also been associated with increased expression of pro- apoptotic and autophagic pathways such as dysregulation on PI3K-Akt pathway and caspase-3^2,4^. As other flaviviruses, ZIKV uses host cell enzymatic machinery and orchestrate a reorganization of lipid metabolism to promote viral replication and processing of its own proteins^5,6^. ZIKV is composed of three structural proteins (capsid (C), precursor membrane (prM), and envelope protein (E)) and seven nonstructural proteins (NS1, NS2A, NS2B, NS3, NS4A, NS4B e NS5)^7^. Among these, NS4A and NS4B were reported as key elements involved in growth restriction through Akt/mTOR stress-response pathway, since their interactions with Akt and mTOR inactivation lead to autophagy^4,7,8^. Markers related to impaired Akt/mTOR signaling have been associated with ZIKV-host interaction and was identified in the serum of infected patients, in a new proposed approach for Zika diagnosis^9,10^.

In addition to neural tissue, the confirmation of ZIKV transmission through sexual contact suggested viral tropism to reproductive tract cells^1,11^. Kumar *et al*. (2018) demonstrated that Sertoli cells are susceptible to ZIKV, and replication is persistent in the male reproductive tract^11^. Moreover, ZIKV was able to infect testis of mice, leading to an important increase in Reactive Oxygen Species (ROS) and consequently to testicular oxidative stress and impaired spermatogenesis^12^. In addition, men infected with ZIKV presented prostatitis and hematospermia, suggesting prostate infection and inflammation^13^. Human prostate epithelial adenocarcinoma (LNCaP) has also shown permissiveness to infection and ZIKV replication, thus confirming cellular tropism to prostate malignant cells. All this data, therefore, suggest human prostate as a ZIKV reservoir on sexual transmission through semen^13,14^.

Given the ZIKV tropism to various cell types, and its ability of interrupting cell growth, a hypothesis was raised about its oncolytic potential. *In vitro*^15,16^ and *in vivo*^17^ experiments were conducted to confirm the antiproliferative activity of Zika against glioblastoma tumor cells. Zhu *et al*. (2017) demonstrated that ZIKV preferentially targets glioblastoma stem cells, differently from another neurotropic flavivirus, the West Nile Virus, WNV, which kills both normal and tumor cells^17^. Due to potential virus virulence latency on CNS^18^, Dabaja *et al*. (2018) evaluated metabolic alterations in glioblastoma cells induced by a ZIKV prototype (ZVp), which also demonstrated cytopathic effects. This prototype based on inactivated ZIKV particles fused with Outer Membrane Vesicles (OMV) from *Neisseria meningitidis* demonstrated significant immune response and great potential for tumor management^16,19^. Additionally to the cytopathic effect demonstrated in glioblastoma cells, ZVp has also shown tropism and antiproliferative effects against the PC-3 androgen-independent human prostate cancer cell line^16^, whose mechanisms are yet to be elucidated.

Prostate cancer etiology, progression and therapy responsiveness have been associated with oxidative stress, DNA instability and aberrant DNA methylation^20,21^. Defense mechanisms against ROS are used as a survival strategy by tumor cells^22^. However, increased ROS, ER stress, cell cycle arrest and DNA damage are also strategies used in anticancer therapy through approaches such as radiotherapy and chemotherapy^23^. One study demonstrated that PC-3 and DU-145 cells differed significantly in their radiosensitivity due to variations in basal and induced Nrf2 (Nuclear Factor Erythroid 2-Related Factor-2) expression levels^24^.This basic leucine zipper transcription factor modulates cell inflammatory and immune response by inducing the transcription of antioxidant enzymes, which have a role in maintenance of cancer cell survival and disease progression^22^. However, despite indications of Nrf2 overexpression in malignant cells^22,25^, several authors demonstrated evidences that cytoprotective enzymes are downregulated in prostate cancer, partially due to hypermethylation of CpG sites in the Nrf2 gene^21,26,27^; thus, the role of Nrf2 on the susceptibility of prostate cancer to oxidative stress remains controversial.

Accordingly, impairments in the cytoprotective activity of Nrf2, blockage of PI3K/Akt/mTOR signaling, and the interaction of ZIKV proteins with key pathways might be used as strategies to increase cancer cell susceptibility to oxidative stress and, consequently, inhibit tumor cell growth^22,25,26,28^. Given the interaction of Zika virus with pathways that play a role on ROS homeostasis^4,8,29^ and promote lipid metabolism modifications^5,30^, we investigated the metabolic alterations induced by ZVp on the PC-3 prostate cancer cell line. Employing a metabolomic approach based on high-resolution mass spectrometry, statistically discriminant biomarkers for PC-3 treated ZVp were selected and structurally proposed as an attempt to correlate the antiproliferative effect reported in the literature with molecular signatures.

## Results

In order to evaluate the metabolic alterations upon inactivated Zika virus exposure, we treated the PC-3 human prostate cancer androgen-independent cell line with ZVp. After 24 hours of incubation, the cellular extracts of non-exposed and exposed cells were directly infused in a high-resolution mass spectrometer for data analysis on positive and negative ion modes.

**Figure 1:**
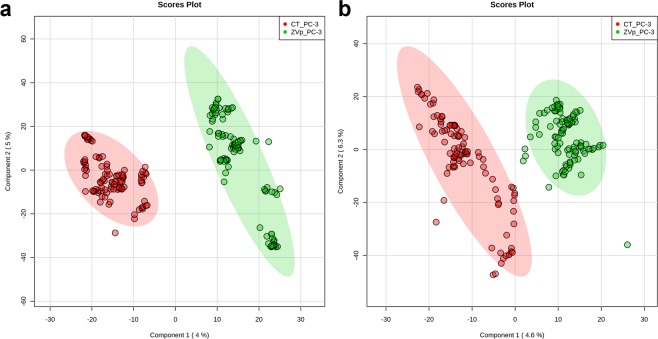
Partial Least Square-Discriminant Analysis (PLS-DA) score plot model showing separation between PC-3 cells control (red) and PC-3 cells exposed to ZVp (green) clustering with data on positive ion mode (**a**) and negative ion mode (**b**).

The acquired mass spectra data were submitted to multivariate statistical analysis (PLS-DA) for group comparison. PLS-DA is a supervised regression analysis, widely used in metabolomics to assess association among sample groups. The principle is based on linear combinations of data variables and further extraction from mass spectrometry raw data features that discriminate sample clustering. Results disposed in Fig. 1 showed remarkable separation between cells exposed to ZVp treatment versus non-exposed cells on both positive and negative ionization modes. The statistical separation among groups confirms the existence of discriminative analytes associated with ZVp-induced metabolic cell alterations. The model was statistically significant on both ion modes (p < 0.001) through validation with prediction accuracy during training permutation test (see Additional File, Fig. S1).

Based on variable importance in projection (VIP) score values greater or equal to 2.2, molecules that metabolically discriminated Group 2 from Group 1 were elected. A heat map analysis (See Additional File, Fig. S2) demonstrated the distribution of the most relevant markers among samples. On the positive ion mode, we were able to identify 10 biomarkers for the ZVp-treated condition, while 11 biomarkers were elucidated on the negative ion mode. Characterization was performed cross-checking data from high-resolution mass spectrometry and metabolomics databases. Proposed chemical structures are described in Table 1.

**Table 1:** Proposed chemical markers elected by PLS-DA VIP scores ≥2.2 from PC-3 prostate cancer cells exposed to Zika virus prototype on both positive and negative ion mode.

Compound	Adduct	Experimental Mass	Theoretical Mass	Error (ppm)	ID1	MSMS
**Positive Ion Mode**						
PGH_2_-EA	[M + K]^+^	432.2505	432.2511	1.39	74984	415, 386, 372
CerP(32:1)^b^	[M + H]^+^	590.4555	590.4544	−1.86	103039	544, 309, 281, 573, 558, 232
N-Acetyl-D-glucosaminyl diphosphodolichol	[M + Na]^+^	610.2145	610.2153	1.31	6251	355, 266, 464, 371, 221, 284
Azelaoyl PAF	[M + H − H_2_O]^+^	634.4436	634.4448	1.89	62938	573, 624, 587, 520
GlcCer (30:1)^b^	[M + H]^+^	644.5105	644.5096	−1.39	7222	612,626, 598, 459, 532, 516
PE(P-32:0)^b^	[M + H − H_2_O]^+^	658.5186	658.5176	−1.52	62174^a^	640, 627, 613, 459
PE(P-34:4)^b^	[M + H − H_2_O]^+^	678.4872	678.4863	−1.33	62157^a^	633, 622, 660, 647, 481/482, 558, 532, 586
PE-NMe(32:0)^b^	[M + H − H_2_O]^+^	688.5292	688.5281	−1.59	40733^a^	671, 566, 431/432, 641, 342, 463, 656
PE(P-32:1)^b^	[M + H]^+^	702.5445	702.5432	−1.85	62178^a^	684, 670, 656, 333, 459, 365, 644
PE(38:5)^b^	[M + H]^+^	766.5394	766.5381	−1.70	60376^a^	720, 706, 747, 734, 548, 569, 485
**Negative Ion Mode**						
HO-PGF_2α_ or HO-PGE_1_^c^	[M + Cl]^−^	405.2042	405.2049	1.73	36117^a^ or 36196^a^	337, 369, 387, 367, 373, 361
Dihomo-PGF_2α_ or Dihomo- PGE_1_ or Isoprostanes^c^	[M + Cl]^−^	417.2408	417.2413	1.19	36206^a^ or 36171^a^	297, 373, 289, 399
Dolichol phosphate D-mannose	[M − H]^−^	425.1939	425.1946	1.64	5925	283,367, 379, 255, 313, 407
PG(14:0)^b^	[M − H]^−^	455.2421	455.2415	−1.31	80000^a^	397, 423, 339, 437, 409,387
PG(12:0)^b^	[M + Cl]^−^	477.1662	477.1661	1.87	4086^a^	415, 459, 433, 417, 409, 441, 449
PA(20:1)^b^	[M + Cl]^−^	499.2606	499.2597	−1.80	82345^a^	383, 373, 311, 261, 431, 441, 481, 453, 463
PI(12:0)^b^	[M − H]^−^	515.2272	515.2263	−1.75	81172	447, 397, 399, 401, 457, 478, 497
5-Methyltetrahydropteroy *l*- tri-glutamate	[M − H_2_O − H]^−^	621.2276	621.2269	−1.13	3684	584, 505, 353, 563, 603, 612
Isocoproporphyrin or Coproporphyrin (I, II, III or IV) ^c^	[M − H_2_O − H]^−^	635.2518	635.2506	−1.89	5665^a^	599, 577, 617
Dehydroisocoproporphyrin	[M − H]^−^	651.2471	651.2460	−1.68	6570	571, 633, 615, 607, 593, 583
Coproporphyrinogen (I or III)^c^	[M + Cl]^−^	695.2863	695.2853	−1.44	63930 or 80	653, 677, 665, 659, 637, 569

^1^METLIN ID; ^a^Representative ID for the class; ^b^Carbon number: double bond; ^c^Not specified-molecules with the same m;z and similar fragmentation profile.

CerP-Ceramide phosphate; GlcCer-Glucosylceramide; PAF-Platelet activating factor; PC-Phosphatidylcholine; PE-Phosphatidylethanolamine; PI-Phosphatidylinositol; PG-Phosphatidylglycerol; HO-PGF_2α_-Hydroxy Prostaglandin F_2α_; HO-PGE_1_-Hydroxy Prostaglandin E_1_; PGH_2_-EA - Prostaglandin H_2_-Ethanolamine.

**Figure 2:**
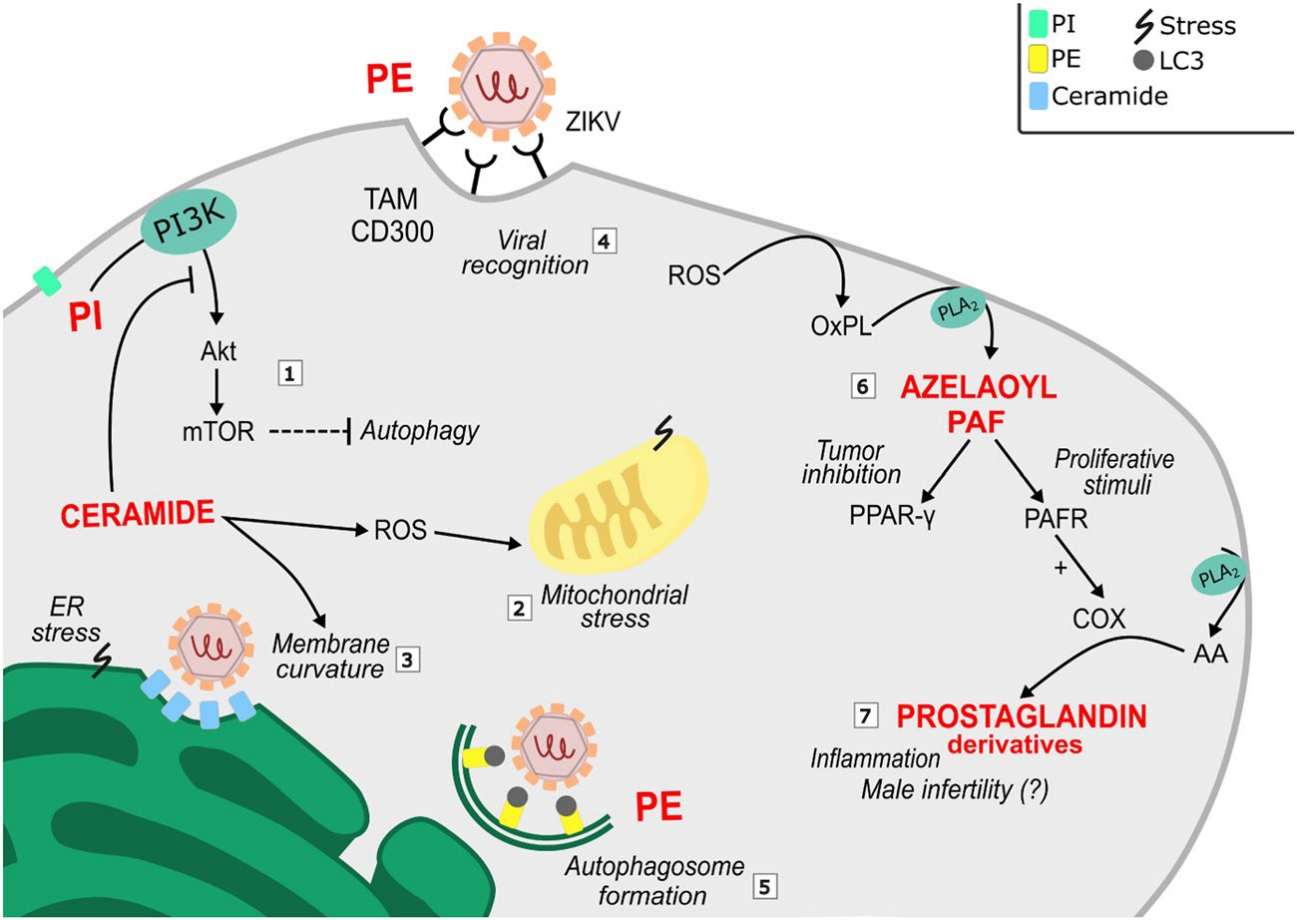
Lipid metabolism alterations induced by ZVp on prostate cancer cells (PC-3 line) mediate increased levels of: phosphatidylinositol, a marker of ZIKV interaction with autophagic pathway (1); ceramides, that contribute to autophagy (1), mitochondrial stress (2) and membrane curvature (3); and phosphatidylethanolamine, which assist on particle recognition (4) and autophagosome formation (5). Zika virus stresses prostate cancer cells leading to high levels of ROS and consequently the presence of oxidized molecules (6) and inflammatory mediators (7). Together, all these factors may contribute to trigger cellular stress and prostate cancer cell death. Abbreviations: AA – arachidonic acid; COX – cyclooxygenase; ER – Endoplasmic reticulum; OxPL – Oxidized phospholipids; PAF – Platelet Activating Factor; PAFR – PAF receptor; PE – Phosphatidylethanolamine; PI – Phosphatidylinositol; ROS – Reactive Oxygen Species.

**Figure 3:**
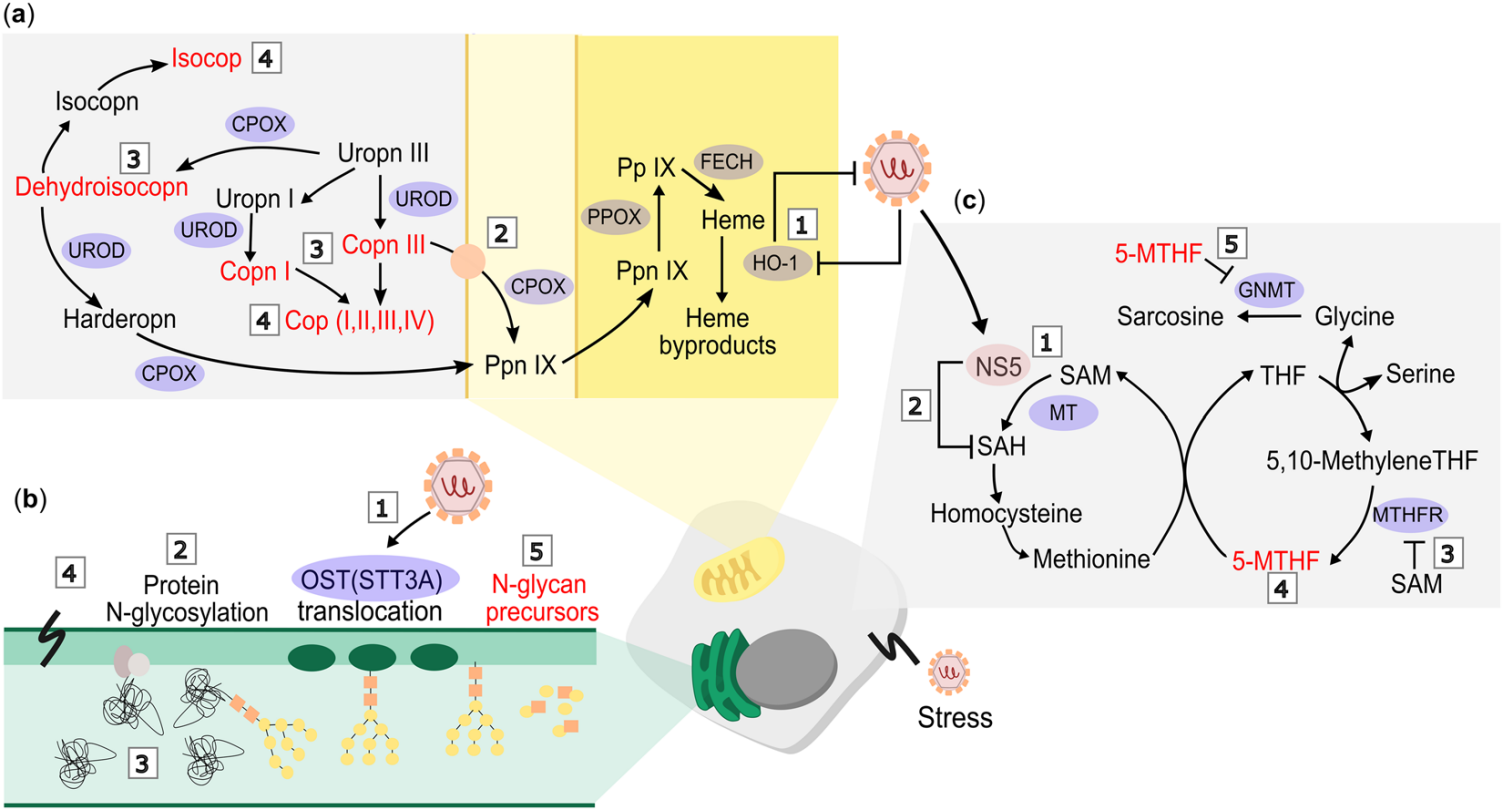
Proposed interaction of ZIKV moieties with pathways involved on (**a**) porphyrin homeostasis, (**b**) protein glycosylation process and (**c**) one-carbon metabolism, leading to the presence of elected biomarkers (in red). (**a**) Heme formation is dependent of an influx of porphyrins to mitochondria which is coordinated by the action of CPOX and UROD enzymes on porphyrin precursors. However, ZIKV interaction with HO-1 enzyme (1) may interfere on heme degradation process. Disturbances on heme metabolism eventually affect porphyrins mitochondrial transport through ABCB6 (2) resulting on porphyrin accumulation (3) represented by Copn (I and II) and Dehidroisocopn and its oxidation products (4) illustrated by Cop (I, II, III and IV) and Isocop; (**b**) ZIKV may interact with OST complex displayed on endoplasmic reticulum (ER) (1), which has an important role on the N-glycan translocation, impairing on host cell protein glycosylation (2). This interaction may lead to misfolded protein formation (3) and ER stress (4) contributing to N-glycan precursors accumulation (5); and (**c**) SAM is an essential methyl donor for methylation processes and its availability impact on the one carbon metabolism molecules abundance. Possible ZIKV NS5 moieties interference on SAM (1) and SAH (2) impact SAM availability and regulation of MTHFR (3), leading to 5-MTHF accumulation (4). As consequence, 5-MTHF negatively control GNMT activity (5), decreasing sarcosine production and promoting a better prognosis to prostate cancer cells. Abbreviations: Cop – Coproporphyrin; Copn – Coproporphyrinogen; DehydroisoPn – Dehydroisocoproporphyrin; HarderoPn – Harderoporphyrinogen; Isocop – Isocoproporphyrin; Isocopn – Isocoproporphyrinogen; Ppn – Protoporphyrinogen; Pp – Protoporphyrin; Uropn – Uroporphyrinogen; CPOX – Coproporphyrinogen Oxidase; UROD – Uroporphyrinogen Decarboxylase; PPOX – Protoporphyrinogen Oxidase; FECH – Ferrochelatase; HO-1 – Heme Oxygenase; OST – Oligosaccharyltransferase; GNMT – Glycine N-Methyltransferase; 5-MTHF – 5-Methyltetrahydrofolate; THF – Tetrahydrofolate; MTHFR – Methylenetetrahydrofolate Reductase; SAM – S-Adenosylmethionine; SAH – S-Adenosylhomocysteine; MT – Methyltransferase.

It was possible to correlate biomarkers’ function with the recent finds on the antiproliferative effect of Zika virus on prostate cancer cell^16^. Some molecules, identified on Table 1, are involved on cell mechanisms impaired during viral exposure, while others suggest altered pathways that might lead to prostate cancer cell death. Among them, we found changes on the lipidomic cell profile of phospholipids and ceramides, inflammatory mediators, N-glycan biosynthesis precursors, porphyrin and folate, whose importance is briefly illustrated in Figs 2 and 3. Supported by literature information, we were able to infer the significance of these molecules on PC-3 prostate cancer cell line metabolism disrupted by viral influence and the role of ZIKV interactions with metabolic pathways of host cells.

## Discussion

Viral replication is a mechanism closely dependent on host cell factors. To achieve this purpose, flaviviruses, including ZIKV, promote a reorganization of lipid metabolism and the endoplasmic reticulum (ER) membrane to favor replication^30^. ZIKV, particularly, promotes alterations on general lipid metabolism^5^. In this context, although the proposed ZVp is a particle with inactivated ZIKV, i.e. there is no actual infection and/or replication, key active or binding moieties from molecules that trigger certain pathways may remain intact, even after thermal inactivation; therefore, some lipid classes may play fundamental roles in the interaction with viral antigens and express mediators of cellular response upon contact with ZVp.

Ceramide phosphate (32:1) *m/z* 590.4555 and Glucosylceramide (30:1) *m/z* 644.5105 were identified as chemical markers for the ZVp-exposed condition. Increased basal levels of ceramides are known to have effects over cell fate regulation, and are also associated with cell stress conditions^31^. During infection of pathogenic flaviviruses (e.g. Dengue Virus (DENV) and WNV), increased ceramide levels have been reported, especially due to their role on viral assembly by membrane vesiculation^32^. Arboleda *et al*. (2009) highlighted the potential of increased of ceramide levels to induce mitochondrial dysfunction and neuronal cells death through autophagy mediated by the PI3K/Akt pathway^31^ (See Fig. 2). Thus, the induction of increased levels of ceramides has been used as an anticancer strategy, given its ability to promote the activation of cancer cell death through apoptosis and autophagic responses^33^.

Other lipids elected by our analysis play an important structural role on cell membranes. Phosphatidylethanolamines (PE), Phosphatidylserines (PS), Phosphatidylinositol (PI), Phosphatidylcholine (PC) Phosphatidic acid (PA) and Phosphatidylglycerol (PG) are abundant lipids on cellular membranes, and therefore are on front of cell-cell and cell-pathogen interactions^30^. Statistically relevant metabolic markers for PC-3 cells exposed to ZVp condition were identified as five PEs with *m/z* 658.5186 PE(P-32:0), *m/z* 678.4872 PE(P-34:4), *m/z* 688. 5292 PE-NMe(32:0), *m/z* 702.5445 PE(P-32:1) and *m/z* 766.5394 PE(38:5) on positive ion mode. Being a product of PS decarboxylation, both PE and PS play roles on ZIKV facilitating viral entry on normal conditions^5^. Furthermore, the increase of intracellular PE positively regulates autophagy in mammalian cells. Autophagic membranes are enriched in PE, and this molecule also participates as an anchor to LC3 autophagic protein. Hamel *et al*. (2015) demonstrated the expressed co-localization of ZIKV viral envelope and cytosolic LC3, suggesting the role of this protein on a ZIKV-induced autophagic process^34^. Thus, the lipidation of LC3 to promote autophagosome membrane formation is a process dependent on PE abundance^35^, suggesting that the excess of PE may contribute to ZIKV-mediated cell death.

Different anionic phospholipids [*m/z* 499.2609 PA(20:1), *m/z* 477.1662 PG(12:0), *m/z* 455.2421 PG(14:0) and *m/z* 515.2274 PI(12:0)] were also elected by the statistical model. Anionic lipids such as PA and lysoPA favor membrane curvature by the action of flipases, and this rearrangement may promote better interaction of membrane phospholipids with flavivirus’ molecules^36^. Phosphatidylinositols in particular had their association with ZIKV previously reported in metabolomic studies^9,16^, corroborating to our findings. When evaluating the cytostatic impact of inactivated ZIKV on glioblastoma cells, Dabaja *et al*. (2018) observed PI and phosphatidylinositol phosphate (PIP) as biomarkers^16^. In addition, Melo *et al*. (2017) reported several alterations on PIP and PIP2 metabolites in the serum of patients infected with ZIKV strain^9^, which suggests that the interaction with ZVp may be sufficient to promote cell alterations. Phosphatidylinositol phosphates are important precursors in PI3K/Akt pathway, which leads to mTORC1 inhibition of autophagic processes and cell survival. The upregulation of this pathway significantly contributes to prostate cancer cell proliferation, migration and invasion. Due to previously reported ZIKV NS4A and NS4B blockage of Akt signaling, the presence of PIs derivatives is expected, and may highlight the molecular signatures of an autophagic fate^4,9,10^.

Cell death may also be triggered by caspase 3 activation in neuroprogenitor cells^2^, a stimulus that may promote generation of platelet-activating factor (PAF) as counteraction for tumor cell repopulation. PAF, nonetheless, presents dual functions: (i) as a contributor to tumorigenesis, and (ii) as a promoter of cell death, participating in a complex regulation process^37^. In ZVp-treated PC-3 cells, a PAF derivative, Azelaoyl-PAF *m/z* 634.4436, was identified as a candidate biomarker. Azelaoyl-PAF is formed by the action of PLA_2_ on oxidized phospholipids (OxPLs) truncated in sn-2 position due ROS damage^38^. Azelaoyl-PAF, an OxPL, stimulates PAF receptor (PAFR) and behaves as an agonist of PPAR-γ, a common receptor on normal tissues and prostate adenocarcinoma cell lines, as well as a reported tumor growth inhibitor. While the interaction with PAFR leads to NF-κB activation and consequently induction of COX and prostaglandins to a pro-inflammatory process and cell resistance^37^, a PPAR-γ agonist may induce prostate cancer growth inhibition^39^.

The PLA_2_ gene has been indicated as a potential therapeutic target in prostate cancer^37,40,41^. Therefore, OxPLs and highly expressed PLA_2_ influence the production of downstream arachidonic acid derivatives, such as prostaglandins by the action of COX enzymes. We found three *m/z* correlated to prostaglandin biomarkers: *m/z* 405.2042 for Hydroxy Prostaglandins F_2α_ (OH-PGF_2α_) or E_1_ (OH-PGE_1_), *m/z* 417.2413 Dihomo Prostaglandins F_2α_, E_1_ or Isoprostane analogs and *m/z* 432.2505 for Prostaglandin H_2_ – Ethanolamine (PGH_2_-EA). On human seminal fluid OH-PGE_1_ and OH-PGE_2_ are the major prostaglandins, with PGE_1_, PGE_2_, PGF_1α_ and PGF_2α_ and OH-PGF also reported^41^. While PGE_2_ promotes cancer development, PGE_1_ exerts antiproliferative activity, which implies that the ratio between these two opposing metabolites is critical to cancer progression^40^. While there is little knowledge about the cellular effects of hydroxylated prostaglandins, some studies tried to correlate their presence on human seminal fluid with spermatozoa motility. OH-PGF is associated to decreased sperm motility while OH-PGE exerts the opposite effect^42^. It has been reported that ZIKV-infected men presented a significant decrease in spermatozoa number and motility, which suggests that Zika virus is a risk factor to impaired male fertility^13,43^. Moreover, the presence of isoprostane analogs from F_2α_ family and E_1_ derivatives as biomarkers indicates extensive oxidative stress, since these molecules are associated with lipid peroxidation in several diseases including prostate cancer^44,45^. Their election as biomarkers suggests elevation on ROS levels in ZVP-treated condition. Altogether, the fine control of prostaglandins, hydroxy prostaglandins and isoprostanes in prostate cancer cell treated with ZVP may be an explanation to male infertility during ZIKV infection^13,42–44^.

Porphyrin derivatives, Isocoproporphyrin or Coproporphyrin isomers (I, II, III or IV) *m/z* 635.2519, Dehydroisocoproporphyrin *m/z* 651.2474 and Coproporphyrinogen isomers (I or III) *m/z* 695.2863 were also found as biomarkers. Free porphyrin metabolites may cause cell damage through oxidative stress; accordingly, the cell engages a well-coordinated heme biosynthesis through heme negative feedback and a fine control of porphyrin derivatives by degradation enzymes and transporters^46^. Heme oxygenase (HO-1) is a stress response enzyme, induced by Nrf2 signaling and inflammation, responsible to transform heme derivatives in biliverdin, carbon monoxide and ferrous iron^25^. HO-1 exhibits antiviral activity against several flavivirus, such as DENV, Hepatitis C (HCV) and ZIKV. However, recently it was observed that ZIKV could limit HO-1 antiviral activity in a translational or post-translational manner, impairing HO-1 efficacy, a real possibility given the role of ZIKV on ER stress^29,47^. Thus, the capacity of HO-1 modulation by ZIKV may implicate in viral persistence and alterations on heme metabolism^29,48^. However, molecules and mechanisms involved in this interaction remain unknown. Lined up with metabolic interference of virus molecules, Nakano *et al*. (2018) discovered that HCV suppresses intracellular protoporphyrin IX (Pp IX), elevates excretion of coproporphyrinogen III (Copn III), higher levels of Pp IX exporter (FLVCR1 and ABCG2) transcription and decreases expression of mitochondrial coproporphyrinogen III importer (ABCB6), altering porphyrin metabolism^48^. Therefore, the observed porphyrins may be resulted from disturbances in heme degradation, biosynthesis and transport. A porphyrin synthesis scheme is simplified in Fig. 3.

Moreover, a fundamental outer membrane transporter of porphyrin to mitochondria for heme synthesis is ABCB6, and its expression responds to intracellular porphyrin levels^49^. The markers elucidate the spontaneous oxidation of Coproporphyrinogens to Coproporphyrin isomers. The presence of these autoxidation products may occur due to porphyrin precursor accumulation. We hypothesize that an impairment on heme degradation through HO-1 by ZIKV molecules may eventually increase mitochondrial concentrations of heme, causing negative feedback on ABCB6 importer, and consequently accumulation of porphyrin precursors. Excess of porphyrin may photosensitize cancer cells, triggering elevation of ROS and cell death^46^. Since this is a complex and important system for cell homeostasis, further experiments need to be addressed to confirm the role of ZIKV on porphyrin metabolism of prostate cancer cells.

Regarding the autophagic process, cytoplasmic vacuolation and ER-derived autophagosome formation is an evidence of response to ZIKV particles^34,47^. The previously discussed lipid remodeling^5^ induced by ZIKV, as well as the excess use of N-linked glycosylation enzymes for cancer cell protein glycosylation are factors that may trigger an ER stress response^30^. N-acetyl-D-glucosaminyldiphosphodolichol *m/z* 610.2145 and Dolichol phosphate D-mannose *m/z* 425.1939, precursors of N-glycan biosynthesis, were identified as biomarkers of ZVp-treated condition. Glycosylation is the most ordinary and versatile posttranslational protein modification, which regulates protein folding and function^50^. The formation of N-glycan structures starts on ER through a series of glycosylation generating the N-glycan core (See Fig. 3). The translocation of N-glycans to translated proteins occurs via the oligosaccharyltransferase complex (OST), composed of STT3A and STT3B subunits^50,51^. Cancer cells present a differentiated pattern of glycosylation through a process highly dependent on glucosyltransferases, ER function and integrity; the importance of this process is such, that protein glycosylation inhibition has been reported as an alternative chemotherapeutic strategy^51^. An OST inhibitor has been reported as a strategy to flavivirus infection blockage, suggesting that ZIKV molecules interact with host cell machinery^6,52^. In fact, ZIKV E protein glycosylation and infectivity is assisted by STT3A expression^52^. Therefore, considering these findings, an increase in N-glycan precursors may appear as a result of ZIKV and PC-3 cell interaction with either N-glycosylation machinery or ER stress. Thus, the interaction between ZIKV and N-glycosylation enzymes, in addition to ROS-mediated ER stress, may lead to the accumulation of misfolded protein and consequently ER function impairment and pro-apoptotic signaling^50^.

Moreover, some ZIKV non-structural proteins interact with host biomolecules on methylation steps^53,54^. The availability of methyl donors requires intracellular folate from a functional one-carbon metabolism^55^. 5-Methyltetrahydropteroyl tri-L-glutamate (5-MTHF) *m/z* 621.2276, a folate derivative, was found as a ZVp-treated biomarker; the same molecule was previously described by Dabaja *et al*. (2018) during glioblastoma antiproliferative assessment^16^, corroborating the importance of this finding. This molecule participates in the conversion of methionine to S-Adenosylmethionine (SAM), homocysteine to methionine, and as downregulation factor for Glycine N-methyltransferase (GNMT)^55^. For instance, 5-MTHF formation is regulated by precursor availability and SAM levels, which in high levels exerts inhibitory effects on MTHF reductase^55^ (See Fig. 3). Coloma *et al*. (2016) reported that the non-structural protein NS5 has homology to methyltransferases and may sequestrate SAM as methyl donor^53^. Moreover, NS5 may bind to S-Adenosylhomocysteine (SAH) blocking the regeneration of homocysteine and consequently remethylation of methionine and SAM^54^. SAM is used in prostate cancer cells for glycine methylation into sarcosine via GNMT; high levels of sarcosine were found to be a biomarker of prostate cancer progression^56^. The inhibition of GNMT results in decreased sarcosine, diminished proliferation and a better prognosis of prostate cancer^56,57^. All these factors may implicate on modifications of intracellular levels of SAM in one-carbon metabolism and, consequently, 5-MTHF accumulation. Furthermore, changes in DNA methylation of genes in developing cells and human neural cells suggest that one-carbon metabolism is a key pathway on microcephaly^58,59^. Given the basal prostate cancer cell aberrant methylation and ZIKV interaction with folate metabolism, ZVp may interfere on DNA instability, thereby controlling cell fate.

All abovementioned biomarkers were proposed to be involved with the effects of Zika prototype on PC-3 prostate cancer cell line; remarkably, they pointed to ZVp-host lipid remodeling and interaction of ZIKV moieties with key pathways for cell homeostasis, namely the regulation of cytoprotective enzymes, autophagic signaling and ROS induced stress, raising new hypotheses about the role of ZIKV as an oncolytic virus and a promoter of male infertility. The metabolomics approach applied herein has allowed not only the observation of a handful of non-obvious biomarkers, but also their related pathways, which are being correlated to Zika for the first time. Our findings suggest, therefore, the involvement of several pathways with the antiproliferative effect of Zika virus against prostate cancer contributing to a promising topic for future research; this also reinforces the importance of studies with broader, untargeted metabolomics/ lipidomics approaches, as most of these pathways might not have been identified by targeted biomolecular assays. Finally, to the best our knowledge, these molecular signatures were the first reported for prostate cancer cells exposed to inactivated ZIKV particles.

## Methods

### Zika virus prototype (ZVp) production

The Brazilian ZIKV strain (BeH823339, GenBank KU729217) was kindly provided by Professor Doctor Edison Durigon (Biomedical Sciences Institute, University of São Paulo). This strain was isolated from an infected patient in 2015 during the Zika outbreak in the State of Ceará (Brazil) and used for ZVp production. *N. meningitidis* (C2135), *Aedes albopictus* (C6/36) and glial cell (M059J) lines were obtained from INCQS—FIOCRUZ (National Institute for Quality Control—Oswaldo Cruz Foundation, Rio de Janeiro, RJ and Cell Bank)

Culture and infection of C6/36 cells with ZIKV strain was used to replicate the virus after cell confluence achieved 70%. C6/26 culture and infection followed the procedure reported by Melo *et al*.^60^. After viral cytopathic effect (CPE) reached 75%, aliquots of 1 mL of ZIKV were stored at −80 °C.

Intending to obtain Outer Membrane Vesicles (OMVs), *Neisseria meningitidis* was chosen as the microorganism to provide OMVs. For that, this bacterial species was grown at 37 °C under 5% of CO_2_ in agar GCB (Difco). *N. meningitidis* OMVs were isolated according to Alves *et al*.^61^ and stored at − 80 °C before use.

The product OMV fused Zika virus was obtained from M059J cells culture. These cells were infected with ZIKV strain, then OMV particles were added and the culture were submitted to agitation, inducing ZIKV-OMV fusion. The supernatants containing OMV-ZIKV fused particle (ZVp) were formed, collected, inactivated at 56 °C for 1 h and characterized for the parameters of size, polydispersity index (PDI), and electric charge potential using a Zetasizer Nano equipment (Malvern Instruments Ltd., Grovewood Road, Malvern, United Kingdom). In addition, a Nano Tracking Analysis (NanoSign Equipment, Malvern Instruments Ltd., Grovewood Road, Malvern, United Kingdom) was used to determine particles per frame and particles per mL (concentration). The tests performed for ZVp characterization and results is described by Martins *et al*. (2018) procedure^19^.

### PC-3 cell culture and ZVp test

PC-3 human prostate cancer cell line was gently provided by Frederick Cancer Research & Development Center, National Cancer Institute, Frederick, MA, USA. Cell cultures growth was performed accordingly to the procedure established by Roman Jr *et al*.^62^. Briefly, 5 mL of RPMI-1640 supplemented with 5% of fetal bovine serum (RPMI/FBS 5% Gibco^®^, USA) and 1% penicillin: streptomycin (Nutricell^®^, Brazil, 1000 U/mL) at 37 °C with 5%CO_2_ was used for PC-3 cells grow until confluence of 80%. For the experiments, PC-3 cells were used between passages 5 to 12.

Tests using PC-3 cell line and Zika virus prototype (ZVp) were similar to those applied for Glioblastoma cells reported by Dabaja *et al*.^16^. Briefly, PC-3 cells were transferred to 96-well plates (100 μL.well^−1^, inoculation density: 4.5 × 10^4^ cell. mL^−1^). To achieve statistical significance, 10 wells were used to culture the PC-3 cells for the control test, and 10 wells to culture PC-3 cells exposed to ZVp at a concentration of 5.9 × 10^7^ ZVp.mL^−1^ (diluted in cell medium) for 24 h at 37 °C and 5% CO_2_. The chosen ZVp concentration, condition, and timepoint were previously determined by GI_50_ data (concentration for 50% of maximal inhibition of cell proliferation) obtained from an antiproliferative assay performed by Dabaja *et al*.^16^. The control group was maintained at the same conditions, except by the exposure to ZVp aliquot.

### Sample preparation

After trypsinization, 200 µL PC-3 prostate cancer cells exposed and non-exposed to ZVp were transferred to a 2 mL plastic tube and stored at −80 °C until analysis. The sample preparation procedure was performed according to Melo *et al*.^9^. Briefly, 20-µL aliquots of each sample were added to a plastic tube containing 200 µL of tetrahydrofuran and vortexed for 30 seconds at room temperature. After agitation, the extract was diluted with methanol (1000 µL q.s.), homogenized and centrifuged at 3200 rpm for 5 minutes for protein precipitation. Subsequently, 20 µL of each supernatant was diluted in 980 µL of methanol and separated in two aliquots of 500 µL each and ionized separately with the addition of 0.1% formic acid (positive ion mode) and 0.1% ammonium hydroxide (negative ion mode) for HRMS analysis.

### High resolution mass spectrometry analysis (HRMS)

Each solution was directly infused in high resolution mass spectrometer ESI-LTQ-XL Orbitrap Discovery (Thermo Scientific, Bremen, Germany) with a nominal resolution of 30,000 (FWHM). Spectral data were acquired using the following parameters: flow rate of 10 μL.min^−1^, capillary temperature at 280 °C, 5 kV spray voltage and sheath gas at 10 arbitrary units. Each sample had 10 technical replicates acquired in the mass range of 400–1100 *m/z* and analyzed using XCalibur software (v. 2.4, Thermo Scientific, San Jose, CA).

### Statistical analysis and structural proposals

To investigate the induced alterations of ZVp on PC-3 cell model a Partial Least Square Discriminant Analysis (PLS-DA) was performed using the online software MetaboAnalyst 4.0 (www.metaboanalyst.ca)^63^. A guided selection of molecules *m/z* is established by the observation of VIP (Variable Importance in Projection) scores, that translate the impact of selected features in the proposed model. Only biomarkers with VIP scores equal to, or greater than, 2.2 were evaluated. Prediction accuracy during training test with 2000 permutations was used to assess model significance on both ion modes. A VIP heat map of the selected markers using the Euclidean’s distance measurement and Ward’s clustering algorithm was built to illustrate the distribution of the most important biomarkers among groups.

The identity of statistically relevant markers were assessed through comparison of high resolution *m/z* marker (mass error <2 ppm) with available metabolomic databases such as HMDB v.4.0 (Human Metabolome database—www.hmdb.ca), METLIN (Scripps Center for Metabolomics, La Jolla, CA) and Lipid MAPS (University of California, San Diego, CA—www.lipidmaps.org). Tandem MS experiments were used for confirmatory purpose, being acquired in the same instrument, using Helium as the collision gas, with energies for collision-induced dissociation (CID) ranging from 20–50 (arbitrary units). Spectra were compared with theoretical mass fragmentation of Mass Frontier software (v. 6.0, Thermo Scientific, San Jose, CA).

## Supplementary information

Supplementary Info

## Data Availability

The datasets used and/or analyzed during the current study are available from the corresponding author on reasonable request.
