# Supplementary material for: Molecular signatures associated with prostate cancer cell line (PC-3) exposure to inactivated Zika virus
Source: Sci Rep. 2019 Oct 25;9:15351. doi: 10.1038/s41598-019-51954-8 (PMC6814752; doi:10.1038/s41598-019-51954-8)
Supplement: Supplementary file 1 — Supplementary Info [file 41598_2019_51954_MOESM1_ESM.pdf]

Molecular signatures associated with prostate cancer cell line (PC-3) exposure to inactivated Zika virus

Jeany Delafiori [1], Estela de Oliveira Lima [2], Mohamed Ziad Dabaja [1], Flávia Luísa Dias-Audibert [1], Diogo Noin de Oliveira [1], Carlos Fernando Odir Rodrigues Melo [1], Karen Noda Morishita [1], Geovana Manzan Sales [1], Ana Lucia Tasca Gois Ruiz [3,4], Gisele Goulart da Silva [4], Marcelo Lancellotti [5], and Rodrigo Ramos Catharino [1,*]

[1] Innovare Biomarkers Laboratory, Faculty of Pharmaceutical Sciences, University of Campinas (UNICAMP), Campinas, São Paulo, Brazil

[2] Medical School, São Paulo State University (UNESP), Botucatu, São Paulo, Brazil

[3] Faculty of Pharmaceutical Sciences, University of Campinas (UNICAMP), Campinas, São Paulo, Brazil

4 Department of Physiological Sciences, Piracicaba Dental School, University of Campinas (UNICAMP), Piracicaba, São Paulo, Brazil

[5] Laboratory of Biotechnology, Faculty of Pharmaceutical Sciences, University of Campinas (UNICAMP), Campinas, São Paulo, Brazil

* Correspondence: RRC - rodrigo.catharino@fcf.unicamp.br

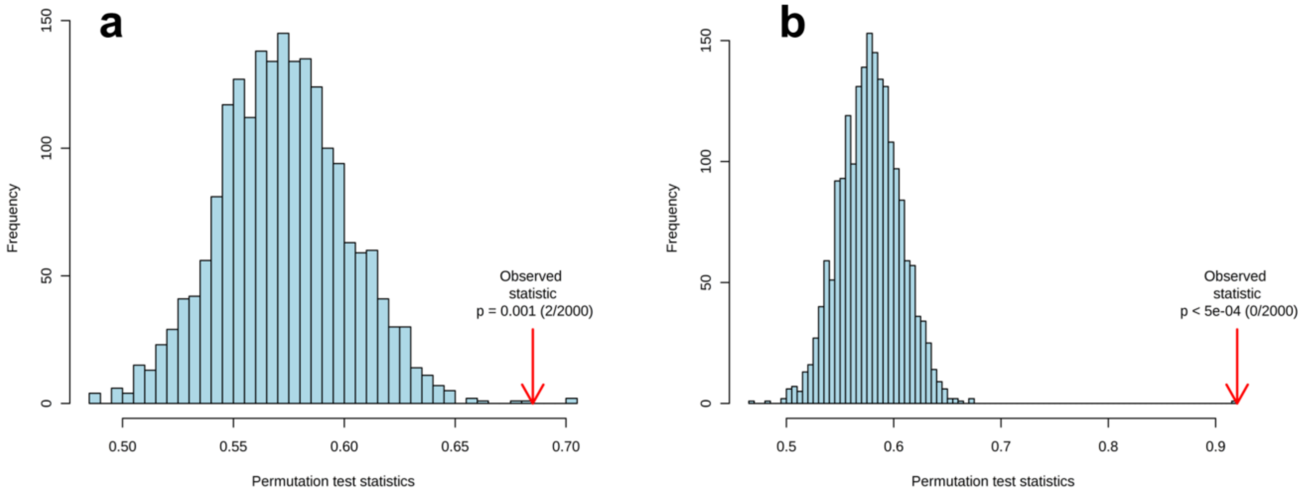

Figure S1: Permutation tests based on prediction accuracy during training for PLS-DA statistical validation. (**a**) p-value < 0.001 (2/2000) on positive ion mode; (**b**) p-value < 5e-04 (0/2000) on negative ion mode.

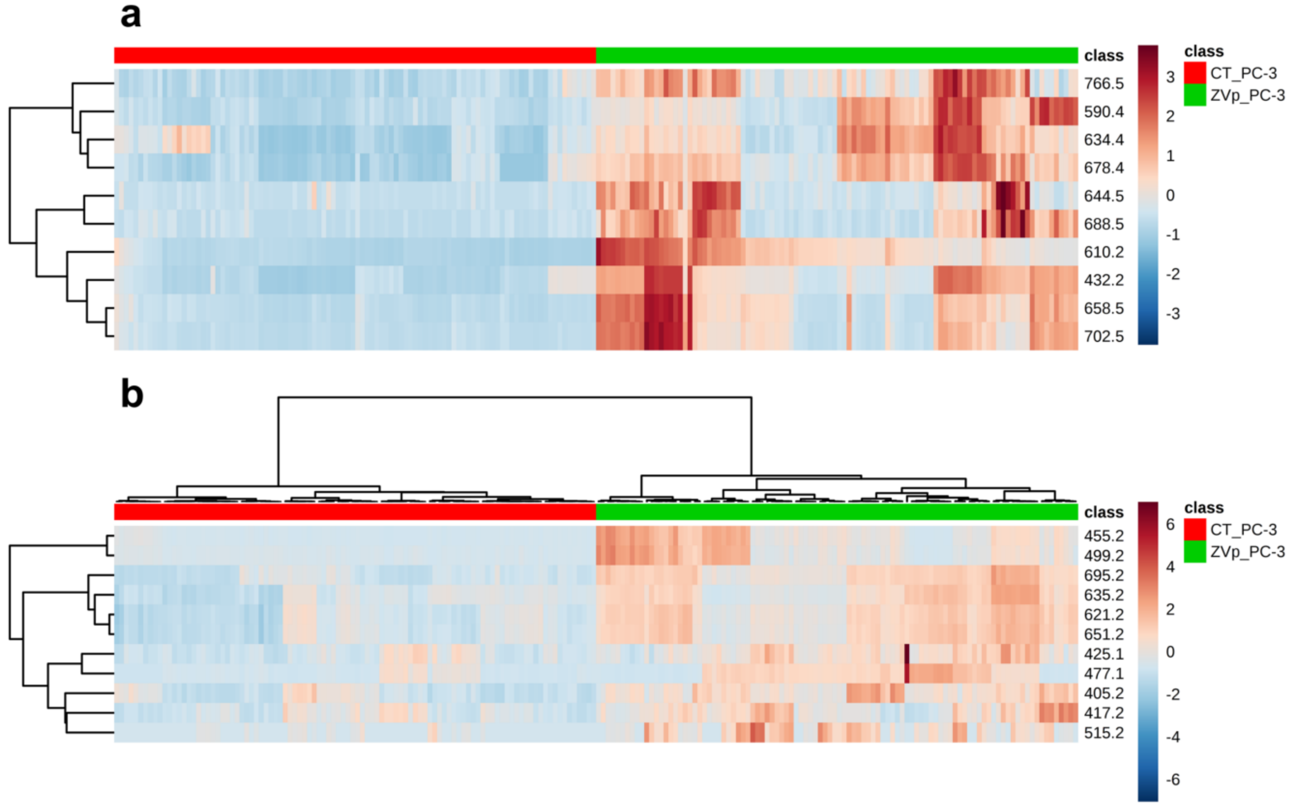

Figure S2: Heatmap analysis of selected markers distribution (**a**) on positive and (**b**) on negative ion mode.
